# Alterations in colorectal cancer virome and its persistence after surgery

**DOI:** 10.1038/s41598-024-53041-z

**Published:** 2024-02-03

**Authors:** Si Xian Ho, Jia-Hao Law, Chin-Wen Png, Rudi Alberts, Yongliang Zhang, Justin Jang Hann Chu, Ker-Kan Tan

**Affiliations:** 1 Laboratory of Molecular RNA Virology and Antiviral Strategies, Department of Microbiology and Immunology, Yong Loo Lin School of Medicine, National University of Singapore, Singapore, 117597 Singapore; 2 Infectious Disease Translational Research Programme, Yong Loo Lin School of Medicine, National University of Singapore, Singapore, 117597 Singapore; 3 Division of Colorectal Surgery, Department of Surgery, National University Hospital, 1E, Kent Ridge Road, NUHS Tower Block, Level 8, Singapore, 119228 Singapore; 4 Department of Microbiology and Immunology, Yong Loo Lin School of Medicine, National University of Singapore, Singapore, Singapore; 5 Department of Surgery, Yong Loo Lin School of Medicine, National University of Singapore, Singapore, Singapore

**Keywords:** Cancer, Microbiology

## Abstract

Viruses are a key component of the colon microbiome, but the relationship between virome and colorectal cancer (CRC) remains poorly understood. We seek to identify alterations in the viral community that is characteristic of CRC and examine if they persist after surgery. Forty-nine fecal samples from 25 non-cancer (NC) individuals and 12 CRC patients, before and 6-months after surgery, were collected for metagenomic analysis. The fecal virome of CRC patients demonstrated an increased network connectivity as compared to NC individuals. Co-exclusion of influential viruses to bacterial species associated with healthy gut status was observed in CRC, suggesting an altered virome induced a change in the healthy gut bacteriome. Network analysis revealed lower connectivity within the virome and trans-kingdom interactions in NC. After surgery, the number of strong correlations decreased for trans-kingdom and within the bacteria and virome networks, indicating lower connectivity within the microbiome. Some co-occurrence patterns between dominant viruses and bacteria were also lost after surgery, suggesting a possible return to the healthy state of gut microbiome. Microbial signatures characteristic of CRC include an altered virome besides an altered bacterial composition. Elevated viral correlations and network connectivity were observed in CRC patients relative to healthy individuals, alongside distinct changes in the cross-kingdom correlation network unique to CRC patients. Some patterns of dysbiosis persist after surgery. Future studies should seek to verify if dysbiosis truly persists after surgery in a larger sample size with microbiome data collected at various time points after surgery to explore if there is field-change in the remaining colon, as well as to examine if persistent dysbiosis correlates with patient outcomes.

## Introduction

Research into the microbiota of patients with colorectal cancer (CRC) has focused predominantly on the bacteriome^1^. While an understanding of CRC-related bacteriome has contributed to disease classification as well as a greater appreciation of the underlying pathogenesis and disease etiology, bacteria represents only a subset of the colon microbiome. Viruses are another key component of the colon microbiome which may contribute to the pathogenesis of CRC that are increasingly recognized^2^. Human specific viruses are known to be associated with cancers more than bacteria due to their mutagenic and functionally manipulative ability^3–6^. For example, the human papillomavirus can cause cervical cancer, and hepatitis C can lead to liver cancer and non-Hodgkin's lymphoma. Viruses could possibly play a bigger role in CRC progression or immune impairment than previously assumed^7^. In particular, bacteriophages make up 90% of the gut virome and serves as the major influencer in modulating the bacterial community composition in patients with CRC^2,8^.

Geicho et al.^9^ found the diversity of gut bacteriophage community to be significantly increased in patients with CRC and identified 22 viral taxa enriched in CRC patients. In particular, members of *Inovirus* and *Tunalikevirus* were found to be enriched in CRC patients. These bacteriophages are known to infect Gram-negative bacterial hosts, such as *Fusobacterium nucleatum* which is implicated in CRC development. Such examples of trans-kingdom microbial interaction mediated by the cancer virome suggests bacteriophages have mechanistic and distinct functions in shaping the bacterial microbiome, and may potentially play an important role in CRC carcinogenesis as well.

The relationship between the virome and CRC appear to be complex at this juncture as there is unlikely a single pathogenic human specific virus that is related to CRC, unlike the direct causal relationship that is established between *Human papillomavirus* and cervical cancer^10^. A few studies have attempted to assess the role of somatic viruses including human papillomavirus (HPV), human polyomavirus, Simian virus 40 and human herpes virus, to carcinogenesis of CRC, most of which have yielded inconclusive results^11–13^. A common finding in the limited existing literature was the dysbiosis of the gut virome that was associated with CRC^8,9^. Dysbiosis refers to microbial compositional changes or imbalances in specific microbial lineages^14^, which in this context alludes to the unique gut microbial profile of CRC patients compared to non-cancer (NC) individuals. The development of CRC appears to be driven by a collective pattern of microbial dysbiosis and interactions, instead of being directly incited by a specific pathogenic microbe, which has not been identified to date .

It is also inconclusive if the state of dysbiosis of gut virome associated with CRC continues to persist after curative surgery. It is postulated that if microbial dysbiosis drives the development of CRC, then resection of the primary tumour should reverse the patterns of dysbiosis associated with CRC and the remaining non-cancer colonic mucosa should exhibit microbiome similar to NC individuals. On the other hand, persistent dysbiosis in the remaining NC colonic mucosa after surgery may imply a pre-existing microbial field change in the colon that possibly predisposed to CRC in the first place.

Bacteriophages are postulated to be a key driver of the carcinogenic bacterial profile, and they serve as key players in modulating the gut bacterial communities associated with CRC pathogenesis. In this study, we seek to examine changes in the viral community (bacteriophage and mammalian specific viruses) in CRC patients before (pre-surgery) and after (post-surgery) operation and to also compare them to NC individuals. Using metagenomic sequencing, we first performed the classical community analysis and statistical tests to compare the gut microbial community structure and composition between CRC patients before and after surgery and NC individuals. Later, we utilized functional network analysis to further explore the differences among the three groups.

## Methods

### Study cohort and fecal sample collection

This was a single center, prospective cohort study conducted between May 2018 to November 2018. Ethics approval was obtained from the National Healthcare Group domain-specific review board (NHG-DSRB), reference number: 2017/01257. All methods were performed in accordance with the relevant guidelines and regulations as outlined by NHG-DSRB, and informed consent was obtained from all subjects. All patients aged 21 and above who were scheduled to undergo elective diagnostic or screening colonoscopy were invited to participate in this study. Patients with pre-existing family history of hereditary non-polyposis colorectal cancer (HNPCC) or familial adenomatous polyposis (FAP), diagnosed inflammatory bowel disease or autoimmune diseases and/or the use of a prolonged course of antibiotics (> 3 days) during the 3 months prior to the study were excluded from study recruitment. Patients who required additional course of antibiotics in addition to routine single dose pre-opreative prophylactic antibiotics were also excluded from the study. All patients received bowel preparation with 2 L of oral polyethylene glycol. Fecal samples were collected from participants 24 h before performing colonoscopy and bowel preparation for both healthy and cancer group. Participants found to have CRC at colonoscopy were assigned to the “Cancer” group while those who did not have cancer and/or adenomatous polyps were assigned to the “Non-cancer” group. An additional fecal sample was collected from cancer group 6 months post-surgery by the patients and mailed to the laboratory within a week. All fecal samples were collected using sterile fecal collection tubes containing 2 ml of RNAlater and stored at − 80 °C until used.

### Sample processing and virome sequencing

Stools were resuspended in RNAlater and total RNA was extracted from the suspension using the protocol as previously described by Zoetendal et al.^15^ with modifications. Two hundred microlitres of stool suspension was added to Precellys 2 mL Soft Tissue Homogenizing Ceramic Beads Kit containing 3 volumes of lysis buffer and 800 ul of acid phenol chloroform. Samples were treated in high-throughput tissue homogenizer at speed 5.5 m/s for 45 s, for a total of 3 times, with cooling interval of 90 s. Samples were centrifuged at 4 °C for 15 min and acid phenol chloroform extraction was conducted on the aqueous phase for a total of 2 rounds and the aqueous phase was retained. 500 μl of chloroform:isoamylalcohol (24:1) was added to the aqueous phase and centrifuged at 13,400*g* at 4 °C for 5 min. One volume of 100% isopropanol and 2 μl of Pellet Paint Co-Precipitant (MilliporeSigma) were used to precipitate the nucleic acid present in the aqueous phase. After centrifugation at room temperature for 5 min at 21,130×*g*, the pellet was washed three times each with 70% ethanol and 100% ethanol. The pellet was them resuspended in 30 μl of 5 mM Tris–HCl (Sigma-Aldrich).

Extracted RNA were fragmented using NEBNext Magnesium RNA Fragmentation Module. Reverse transcription was performed using Maxima H Minus Double-Stranded cDNA synthesis and purified with Nucleospin Gel and PCR Clean-up kit (Macherey–Nagel). Library was prepared using KAPA Hyper Prep kit and NimbleGen adapter kits. The quality and quantity of prepared library were measured using Qubit 2.0 and Agilent Bioanalyzer DNA 100 assay (Agilent Technologies). Viral sequences were further enriched using probe capture-based system as previously described by Ho et al.^16^. Captured libraries were amplified and purified before sequencing on NovaSeq 6000s in 2 × 150 paired-end format.

Paired-end FASTQ files generated from sequencing were analyzed with Genome Detective (https://www.genomedetective.com/) for quality control and pre-processing using default parameters of the platform. This includes removal of adapters and filtered to exclude human and bacterial reads. Background contamination sequences were removed through comparison with in-house database generated from the sequencing of mock communities. Mock communities were generated by combining purified laboratory stocks of coxsackievirus A6, coxackievirus A16, chikungunya, dengue virus 2, and Zika virus.

### Statistical analysis

Alpha diversities (Shannon’s diversity, Chao1 richness, Simpson’s diversity) and Bray–Curtis distancing were computed using vegan packages^17^. Statistical significance between groups were determined using Kruskal–Wallis test^18^. Non-Metric Multidimensional Scaling (NMDS) ordinations based on Bray–Curtis distances were calculated using the R package vegan and plotted using ggplot2^19^. Co-occurrence relationships within viruses and between viruses and bacterial kingdom were estimated using SparCC algorithm and association network graph was generated using igraph packages in R^20^. The concept of connectivity is a well-established and widely used concept in microbiome network analysis, and refers to the degree to which the individual nodes of the network are connected to other members of the microbiome^9^. LEfSe was used for linear discriminant analysis^21^.

## Results

### Study population

Fecal samples were obtained from 12 patients with CRC and 25 NC controls. All surgeries were performed laparoscopically and defunctioning ileostomy was performed for the 2 patients who underwent low anterior resection. None had morbidity which was Clavien grade 3 and above. The participant and clinical data are summarized in Tables 1 and 2, respectively. Using T statistics (non-centrality parameter) power calculation, the number of samples included in this study provide sufficient power to detect effect size of 2, at 95% confidence and 80% power^22^. Only results with LDA ≥ 3.5 and magnitude of correlation > 75% were reported as statistically significant. For CRC patients that underwent surgery, an additional fecal sample was collected 6 months post-operation. To investigate if CRC influences the gut microbiome, fecal virome of patients with CRC were compared with healthy controls. Fecal samples were subjected to metagenomic sequencing and viruses were enriched using a probe capture-based system. On average, 79,733,645 raw reads were generated per sample and 79,298,068 valid sequences have remained for analysis after filtration for low-quality sequences. The number of reads per sample is plotted to analyse inter-individual variation (Supplementary Fig. 1). Relative abundance for each of the virus species was calculated using the number of reads assigned to the virus divided by the total number of reads generated in each fecal sample.

**Table 1:** Overview of participant clinical characteristics.

	Cancer (% or SD) n = 12	Healthy (% or SD) n = 25
Age, mean	63.8 (± 9.3)	61.6 (± 8.9)
Gender		
Male	7 (58.3%)	14 (56.0%)
Female	5 (41.7%)	11 (44.0%)
Body mass index, mean	25.8 (± 4.0)	25.2 (± 4.4)
Adjuvant chemotherapy	7 (58.3%)	–

**Table 2:** Clinical data of cancer group patients.

Subject	Age	Gender	Race	Tumor location	TNM stage	Surgery
M02-P	57	Male	Malay	Sigmoid	3	Anterior resection
M03-P	74	Female	Chinese	Descending	3	Left hemicolectomy
M04-P	56	Female	Malay	Sigmoid	1	Anterior resection
M06-P	60	Female	Malay	Sigmoid	3	Anterior resection
M07-P	67	Male	Chinese	Sigmoid	2	Anterior resection
M08-P	46	Male	Chinese	Rectum	3	Anterior resection
M09-P	59	Female	Indian	Rectum	2	Anterior resection
M11-P	65	Male	Chinese	Sigmoid	2	Anterior resection
M12-P	65	Male	Indian	Rectosigmoid	1	Anterior resection
M13-P	69	Male	Chinese	Sigmoid	3	Anterior resection
M15-P	82	Female	Chinese	Rectosigmoid	3	Anterior resection
M16-P	66	Male	Chinese	Sigmoid	1	Anterior resection

**Figure 1:**
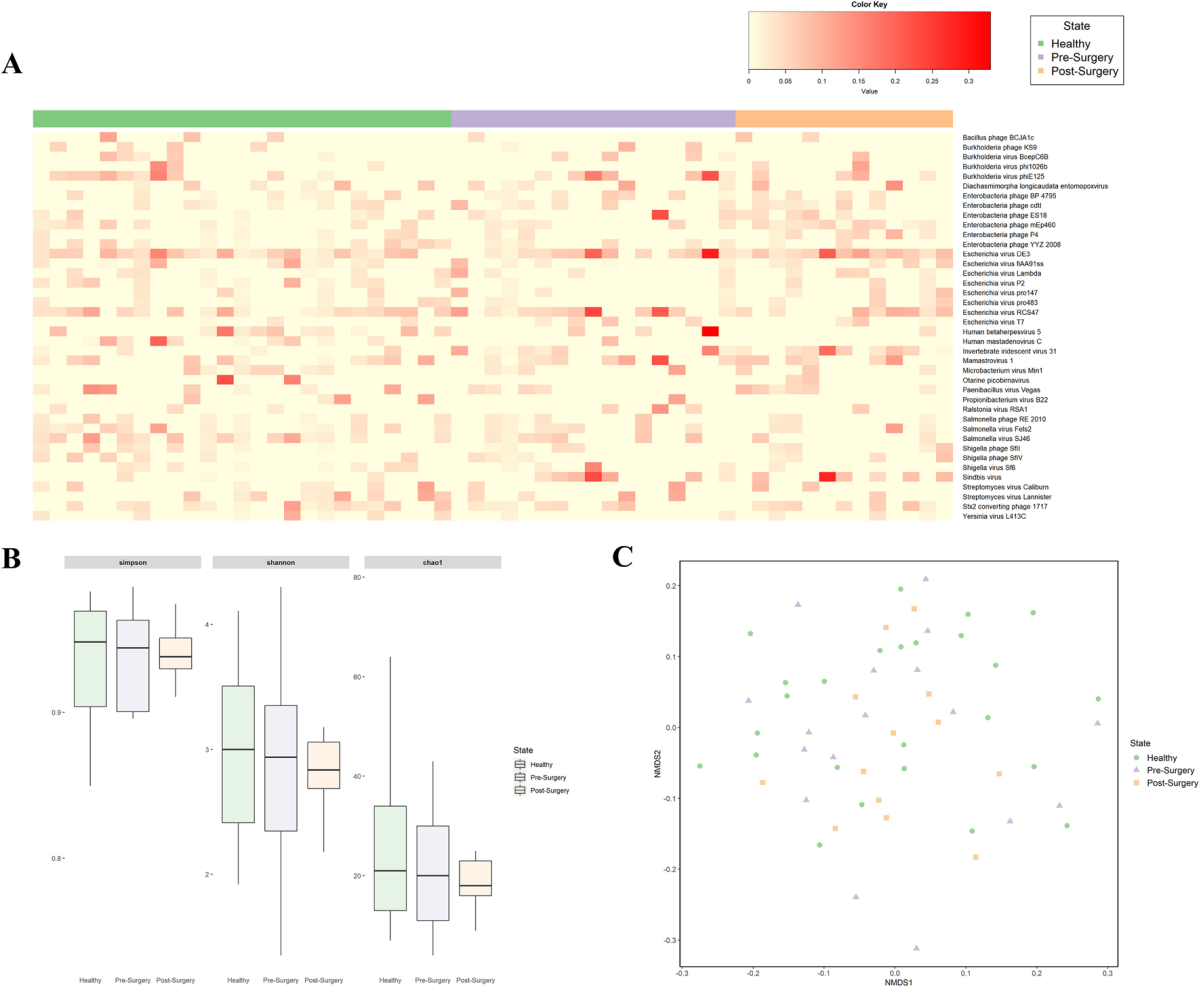
Virome composition of fecal samples obtained from healthy and CRC patients (pre-surgery and post-surgery). (**A**) Heatmap of the top 40 relative abundance of viruses in healthy, pre-surgery and post-surgery samples. (**B**) Comparison of alpha diversity between healthy and CRC patients. Viral diversity is based on Shannon and Simpson diversity and viral richness is based on Chao1 index. (**C**) NMDS plot using Bray–Curtis distancing method.

Of the top 40 viruses detected in the fecal samples, the majority of them were found to be bacteriophages, with only 4 vertebrate viruses detected (Fig. 1A). Among them, Mamastrovirus 1, which is associated with acute gastroenteritis in children, was detected in 41.8% of the fecal samples. Other human-associated viruses detected were human betaherpesvirus 5 and human mastadenovirus C which were less prevalent in the fecal samples as compared to mamastrovirus 1.

### Virome community is altered in CRC patients compared to NC individuals

**Figure 2:**
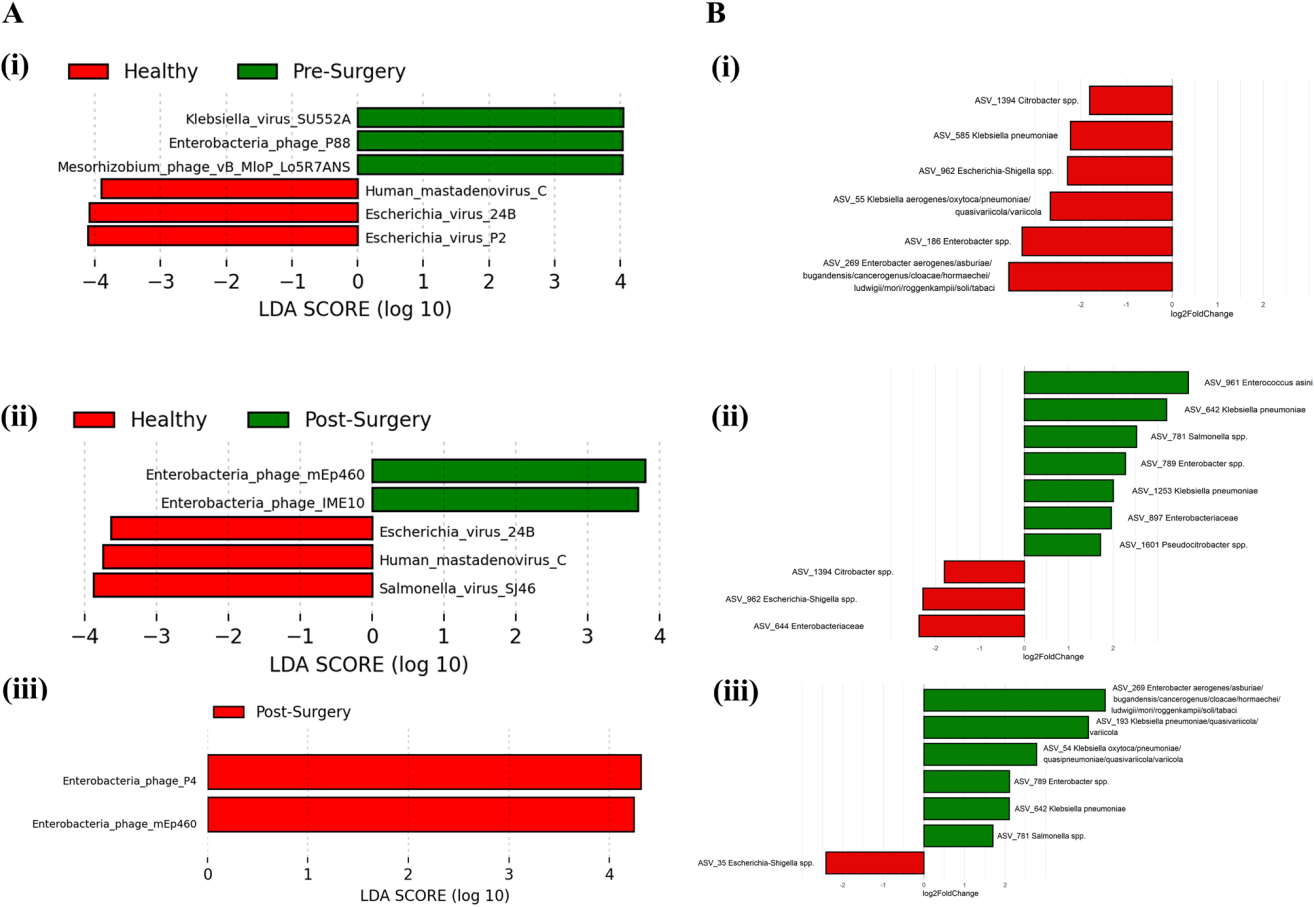
(**A**) Linear discriminant effect size analysis (LEfSe) showing viruses (LDA > 2.0) that account for the difference between (i) healthy and pre-surgery, (ii) heathy and post-surgery, and (iii) pre-surgery and post-surgery. (**B**) Log2fold change in the relative abundance of ASVs of (i) pre-surgery over healthy, (ii) post-surgery over healthy, and (iii) pre-surgery over post-surgery.

Similar alpha diversity was observed among the three groups (Fig. 1B). While post-surgery tends to have lower evenness and richness as measured by Shannon, Simpson and chao1 indices, this difference was not significant using ANOVA. There was also no clear distinction among the three groups as evaluated by ordination plot NMDS using Bray–Curtis distance (Fig. 1C). Despite that, discriminative features could be determined using the LEfSe analysis when compared between healthy and pre-surgery, healthy and post-surgery, and pre-surgery and post-surgery (Fig. 2A) groups. Majority of the discriminative viruses identified were bacteriophages. Human Mastadenovirus C and *Escherichia* virus 24B were the key signatures of a healthy profile as compared to patients with colorectal cancer (pre-surgery) and continues to be the key signatures that differentiates the between the non-cancer and CRC patients post-surgery. Two of the *Enterobacteria* phages (Enterobacteria phage P4 and Enterobacteria phage mEp460) were the discriminative species that were enriched in the fecal sample of the CRC patients post-surgery relative to pre-surgery.

To determine the impact of enriched or depleted bacteriophages on the bacterial community, the alteration in the bacteriome for each cohort were also analyzed. Data on the bacteria community was obtained from bacteriome study previously conducted on the same subjects. With mainly Enterobacteriaceae phages identified as the key signatures of the healthy and pre-surgery cohort, a reduced level of Enterobacteriaceae bacteria was also observed in the pre-surgery cohort (Fig. 2B). Similarly, on comparison between the post-surgery and heathy cohort, the depletion of *Salmonella* virus SJ46 in post-surgery coincides with an increased in *Salmonella* spp. However, the relationship for other top differentially abundant microbial signatures were less distinct. One thing to note for these comparisons is that most phages have limited host ranges and would generally only infect one bacterial species^23^. Given the low resolution of 16S rRNA sequencing, species cannot be determined for some of the ASVs. Rather than examining the relationship between phages and its bacteria host, we seek to provide an overview of the co-occurrence signature between the microbes and understand the dynamics between possible interaction that is beyond the abundance of individual virus or bacteria using network analysis.

### Increased viral correlations and network connectivity in CRC patients compared to NC individuals

**Figure 3:**
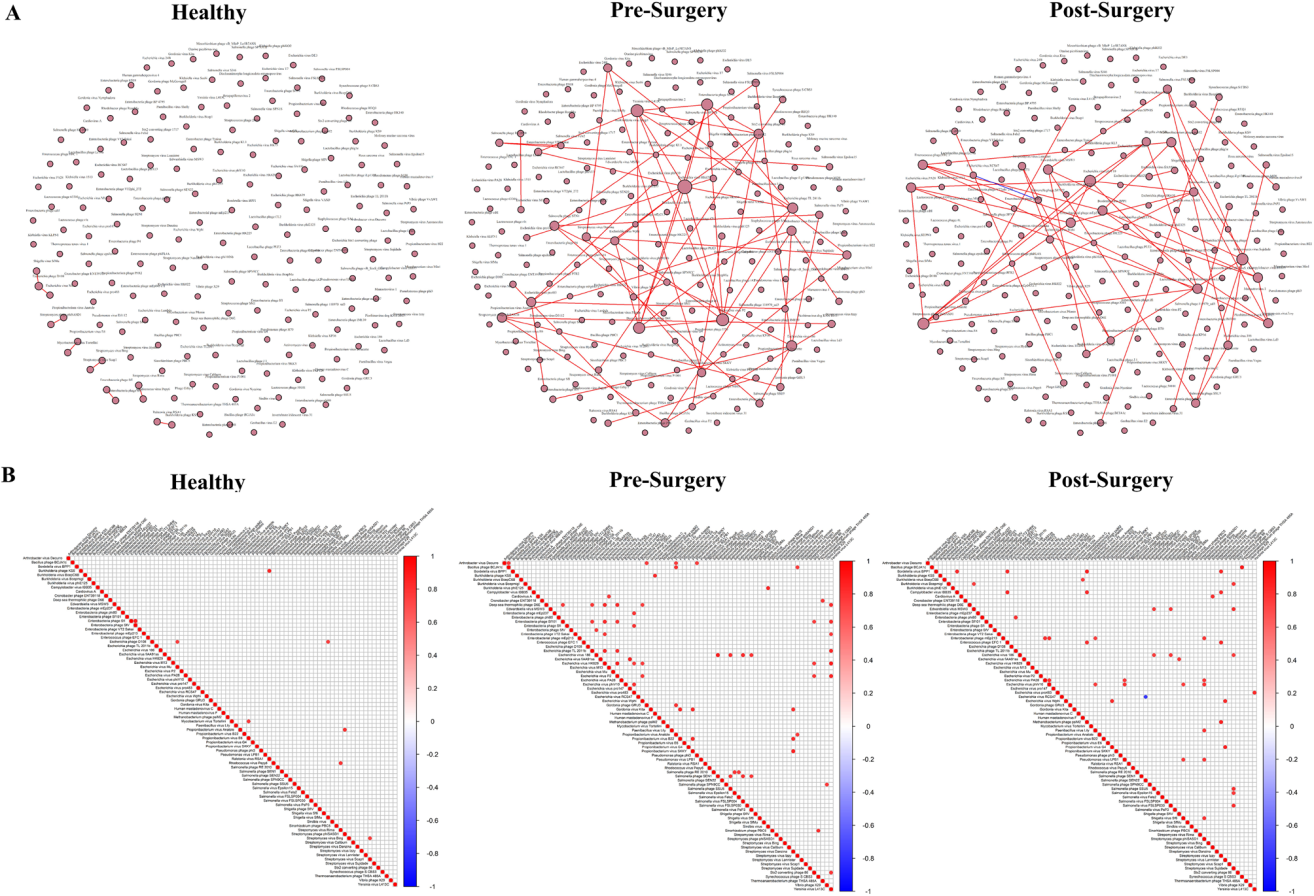
Network analysis of fecal microbiota using SparCC correlation coefficients showing (**A**) virus-virus correlation network in healthy, pre-surgery and post-surgery. Each node represents a virus species, and the edges represent the correlation coefficients between the viruses with blue edges representing co-exclusion and red edges representing co-occurrence. (**B**) Heatmap of SparCC correlation coefficients between viruses in healthy, pre-surgery and post-surgery populations. Only correlations with a magnitude higher than 0.7 were shown.

Co-occurrence network analysis was conducted to explore potential symbiotic relationships between viruses using SparCC and the network was modeled as undirected graph consisting of nodes representing each virus and edges representing their co-occurrence or co-exclusion relationship (Fig. 3A,B). 99 significant co-occurrence and co-exclusion relationships were observed in total. Alteration in the number of significant correlations between viruses was observed for colorectal cancer patients (pre-surgery and post-surgery). Positive co-occurrences were mostly observed in all cohort networks, with the exception of 1 negative co-occurrence between *Escherichia* virus RCS47 and *Enterobacteria* phage phiP27 in the pre-surgery cohort. In healthy controls, correlations between viruses are mostly independent with only 1 shared correlation observed. A shift in co-occurrence was observed in colorectal cancer patients (both pre- and post-surgery) where viruses with a higher number of correlations were observed, with five viruses co-occurring with at least four other viruses.

Degree centrality was computed to identify potential hubs and virus clusters that are likely to have a greater influence on the microbial ecosystem (Supplementary Fig. 2). The normalized degree centrality was significantly higher in pre-surgery when compared to healthy, suggesting increased connectivity between virome in patients with colorectal cancer. This is also observed when comparing post-surgery cohort to the healthy cohort. There is no significant difference observed in network connectivity after surgery (post-op) as compared to pre-surgery even though the co-occurring clusters were different. The biggest cluster in pre-surgery group consisted of 13 viruses and was mainly dominated by *Enterobacteria* phages (30.77%) and *Escherichia* phages (46.15%) while the largest cluster in post-surgery group consisted of only 7 viruses and was mainly dominated by *Salmonella* phages (42.86%). *Escherichia* virus HK629 was the largest hub in pre-surgery group, co-occurring with 9 other viruses. In post-surgery group, *Escherichia* virus phiV10, *Streptomyces* phage phiSASD1, and *Streptomyces* virus Sujidade were identified as the largest hub in the cluster, with each member co-occurring with 6 other viruses.

### Unique alterations in trans kingdom correlation network in CRC patients compared to NC individuals and their changes after surgery

Trans kingdom interplay between viruses and bacteria could directly or indirectly impact the viral composition through infection of their host or by modulating the environment. To assess the trans kingdom interaction, the bacteria community of the microbiome was also investigated.

**Figure 4:**
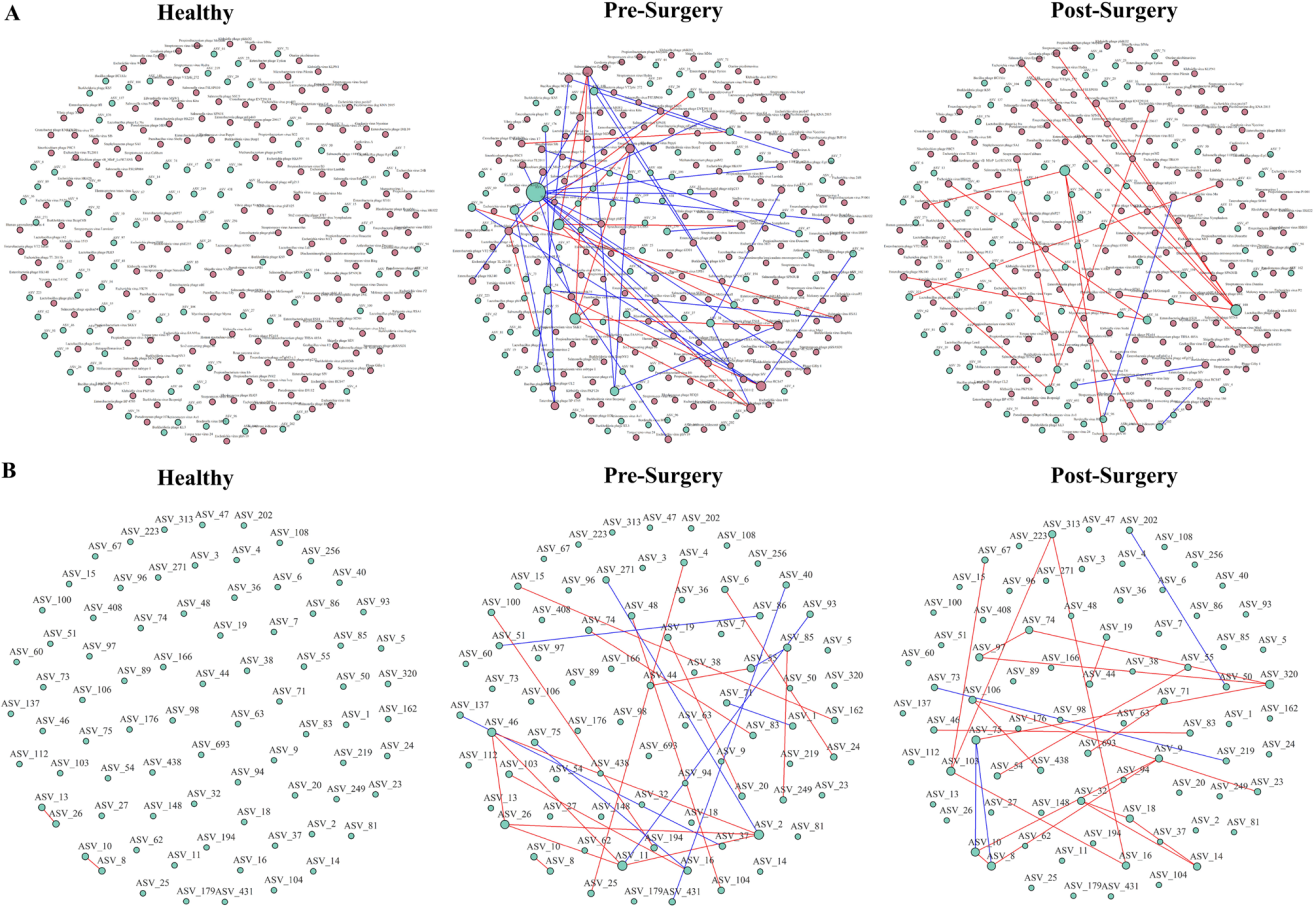
Network analysis of fecal microbiota using SparCC correlation coefficients showing (**A**) trans kingdom interaction between virus-bacteria with at least 0.7 in magnitude and (**B**) bacteria-bacteria correlation network correlation in healthy, pre-surgery and post-surgery. Each node represents a bacterial ASV or virus, and the edges represent the correlation coefficients between the ASV with blue and red indicating co-exclusion and co-occurrence, respectively.

In pre-surgery, negative co-occurrence dominates the trans kingdom network, however, after surgery, positive co-occurrence dominates the network. The number of strongly correlated networks (> 0.75 or < -0.75) were also reduced post-surgery, from 49 trans kingdom co-occurrence to 20 (Fig. 4A). None of the trans kingdom co-occurrence patterns were maintained post-surgery.

Enterobacteria phage P88, which was identified as one of the discriminative signatures in the pre-surgery cohort that differentiate from the healthy cohort, was shown to be positively correlated to *Megasphaera* spp. (ASV 10 and ASV 8) but negatively correlated to a butyrate-producing bacteria, ASV 60 (*Anaerostipes hadrus*).

The virus that displayed the most transkingdom co-occurrence in the pre-surgery cohort, *Salmonella* virus Epsilon15, showed the same co-occurrence patterns as Enterobacteria phage P88 with an additional negative co-occurrence with ASV 5 (*Romboutsia_ilealis/timonensis*). After surgery, co-occurrence between *Salmonella* virus Epsilon15 and *Megaspharea* spp. was lost, instead, it was shown to be positively co-occurring with an unknown species of *Anaerostipes* (ASV 100).

A similar reversion of trans kingdom co-occurring patterns of *Enterobacteria* phage mEp460, a key signature of the post-surgery cohort, was also observed. Prior to surgery, *Enterobacteria* phage mEp460 was negatively correlated to ASV 60 (*Anaerostipes hadrus*). But after surgery, it regained co-occurrence with ASV 60.

Conservation of co-abundance of bacteria under the genus of *Megasphaera*, ASV 10 and ASV 8, was observed in all three cohorts **(**Fig. 4B**)**. In post-surgery, this conserved pair showed additional co-occurrence with ASV 9 (*Faecalibacterium prausnitzii*) and co-exclusion with opportunistic pathogen ASV 75 (*Intestinibacter bartlettii*). Alterations in the bacteriome network were also observed for ASV 75 from pre-surgery to post-surgery. In pre-surgery, ASV 75 showed a negative correlation with an unknown species of *Streptococcus* sp. (ASV 16) but this correlation is lost in the post-surgery cohort and instead becomes negatively correlated to the conserved pair (ASV 10 and ASV 8) and forms an additional correlation with ASV320.

Another notable observation is that in pre-surgery, *Escherichia* virus phiv10 is negatively linked to one of the main butyrate producers in the intestine *Faecalibacterium prausnitizii* (ASV 9), which was suggested to reduce the occurrence CRC and inflammatory bowel disease^24,25^. ASV9 was also a member of the largest cluster in the post-surgery bacteriome network, consisting of 6 other members. *Escherichia* virus phiV10 became the largest virus hub post-surgery and was surprisingly found to be positively co-occurring with ASV 37 (*Ruminococcus* torques group NA) which is a bacterial species that is associated with inflammatory diseases and inflammatory bowel disease^26^ and is known to decrease gut barrier integrity^27^.

In the CRC cohort, we also observed co-occurrence between ASV_103 (*Streptococcus mitis/parasanguinis*) and ASV_16 (*Streptococcus* sp.) that remains stable even after surgery, suggesting that while surgery causes a shift in the microbiome network interactions, there is still a portion of the altered microbiome which persists.

## Discussion

Gut microbial dysbiosis has been found to provide opportunity for the proliferation of oncogenic bacteria^28^. Many studies on the gut microbiome have identified potential bacterial species that play a role in CRC carcinogenesis by driving the initiation and progression of CRC through immune dysregulation^29–31^. However, to date, none of the bacterial species identified were shown to possess sufficient virulence to act alone in causing the disease. This may suggest that different combinations of microorganisms act in synergy to possibly cause a change in the host environment which ultimately leads to the initiation or pathogenesis of CRC. In this study, we have explored the neglected virome community of the gut microbiome in CRC patients and healthy individuals and focused on the interaction between gut bacteriome and virome to provide insights into trans kingdom crosstalk between both groups. In addition, the virome of the fecal samples obtained post-surgery was also investigated to observe if dysbiosis persists after surgery.

Several enriched bacterial strains have been found to be associated with CRC, with the most consistently reported ones being *Bacteroides fragilis* and *Fusobacterium nucleatum*^32^. On the other hand, existing literature on the alterations in virome in CRC patients has been conflicting. Nakatsu et al. found enrichment of Orthobunyavirus in fecal samples of CRC samples as well as enrichment of bacteriophages that are known to infect Gram-negative CRC-associated bacteria^9^. However, another study by Hannigan et al.^8^ proposed that phage communities do not directly modulate the influential bacteria population as the study did not find a strong correlation between the relative abundance of influential phages and specific bacteria. Instead, it was suggested that influential phages advance carcinogenesis indirectly by infecting a broad spectrum of bacteria in the microbiome. This results in changes in the entire bacterial community composition and dynamics, therefore promoting the development of cancer.

In our study, typical virome analysis pipelines which include beta and alpha diversity could not distinguish between the healthy, pre-surgery, and post-surgery cohort. Likewise, Hannigan et al. reported no significant differences between virome of healthy and cancerous state using basic diversity metrics. Instead, they utilized machine learning methods to successfully show the alteration in gut virome for patients with CRC^8^. We explored the relationships within the virome and observed increased connectivity in the cancer cohort (pre-surgery and post-surgery) as compared to the healthy cohort, suggesting that the virome community was indeed altered. Within viruses themselves, most of the co-occurring patterns observed were positive. This is in line with another study that studied the co-occurrence networks of five other gut-related diseases and found that there are lesser negative correlations between viruses as compared to virus-bacteria correlation^33^. The study suggested that viruses rarely antagonized each other but may limit the bacteria population within the microbiome. This reinforces the need to look beyond just within virus-virus network and bacteria-bacteria network.

Co-exclusion of influential viruses to bacterial species that are known to be associated with healthy gut status was observed in the pre-surgery cohort which suggests a possibility of influential viruses disrupting the healthy gut microbiome, resulting in susceptibility to the development of CRC. There was a reversal of the co-occurrence relationship between Enterobacteria phage P88 and Enterobacteria phage mEp460 with *Anaerostipes* sp. after surgery. Prior to surgery, both Enterobacteria phages negatively co-occurred with *Anaerostipes* sp., while in the post-surgery cohort, there was a positively co-occurring relationship between the 2 organisms. Ai et al. observed that the abundance of *Anaerostipes* was significantly higher in the healthy cohort than in CRC samples^34^. Wu et al. also found significant negative correlations associated with *Anaerostipes* in the co-occurrence network of differential ASVs between adenoma and normal cohort^35^. *Anaerostipes* is known to be able to utilize the glucose and fermentation intermediates acetate and lactate to form butyrate and hydrogen, and is thus considered an important microbe in maintaining intestinal metabolic balance and may protect against colon cancer by its production of butyric acid^36^. Enterobacteria phages were also found to dominate healthy controls and decreased along with CRC development^37^.

We hypothesized that surgery should greatly influence the composition of the intestinal microbiome and virome since a large mass of cancer cells was removed during surgery. This also removes the interaction between the cancerous cells and the members of the microbiome. We postulated that the virome post-surgery would gradually resemble that of a healthy individual. As observed in the healthy cohort, network analysis revealed lower network connectivity within the virome as well as transkingdom interactions. After surgery, the number of strong correlations decreases for both trans kingdom as well as within the bacteria and virome network, suggesting a possible reversion to the healthy state with lower connectivity within the microbiome. We observed new positive associations between influential viruses with bacteria associated with a healthy gut status (*Anaerostipes* sp.) in the post-surgery cohort which seems to further support the hypothesis of reversion.

However, the initial 2 positive associations with other healthy gut-associated bacteria (*Megasphera* spp.) were lost. In addition, positive associations with bacteria that are associated with diseased gut such as IBD were observed in the post-surgery cohort. It is likely that dysbiotic patterns continue to persist after surgery as surgery does not completely remove all viable cancer cells since cancer cells could be exfoliated from the primary tumor and continue to reside within the gut. Another possibility is that in this study, the microbiome post-surgery was determined only 6 months after surgery which might not be sufficient for the microbiome to be restored back to a healthy status. There was no consistent trend in the alteration of network connectivity when comparing healthy individuals to pre-surgery, as well as from pre-surgery to post-surgery. Both the emergence of new connections and the loss of co-occurence were observed in the network connectivity between members of the microbiome. This is similar to a study conducted by Nakatsu et al., in which weighted eigenvector centrality scores were compared among bacteriophages from healthy individuals, as well as those in early and late stages of CRC^9^. The findings of the study revealed a significant increase in centrality scores for certain bacteriophages while some bacteriophages species resulted in a loss of connectivity. Nevertheless, a shift in the network connectivity was still observed. This suggests that there were changes in the interactions and relationship among nodes within the network, which could have resulted from various factors such as progression of the disease, the surgical procedure, or other factors. These changes may have considerable significance in understanding the underlying mechanisms of the network and the dynamics of the biological system it represents. The exact mechanism behind the shifts in virome profile after surgery remain to be elucidated and may be contributed by various factors such as alterations in the tumour microenvironment, direct removal of the tumor burden or changes in anti-tumour immune activity. The results of our study lay the groundwork for future research to answer this important question. Alterations in bacteriome-dependent immunological responses in CRC in both the innate and adapative immune system have been described^38,39^. However, studies in virome associated changes in anti-tumour immune mechanisms remain lacking. Understanding how oncogenic virome profile is altered can potentially aid us in developing diagnostic and therapeutic tools for CRC. Unique signatures in immune cell can potentially be utilized to develop an immune cell panel for early diagnosis of CRC. On the other hand, therapeutic interventions may be able to optimize virome profiles to amplify responses to cancer immunotherapy.

Our study has a number of limitations. All recruited CRC patients incidentally had left-sided CRC. As right and left-sided CRC are known to have different molecular biological characteristics and potentially possess different microbial profiles, the results of our study may not be applicable to right-sided CRCs. Microbial analysis in this pilot study was based on stool samples, which is postulated to reflect the cancer microbiota due to downstream shedding of cancer cells. Although the analysis of stool samples might not fully reflect the altered microbiome in CRC tissues, it serves as a representative sample as evidenced by various studies in existing literature which similarly examined stool samples and reported significant differences in bacteriome between CRC and NC individuals. Differences in diet and adjunct treatment such as chemotherapy and radiotherapy may influence gut microbiome and be potential confounders in our study. Therefore, the results of our study should ideally have accounted for differences and changes in dietary patterns. Future studies with larger sample sizes should perform subgroup analysis to explore how adjunct treatments affect gut microbiome and influence dysbiosis. In terms of methodology, while sequencing method is unable to distinguish between active, dead, and dormant microbes, it is a widely used and an effective method for characterizing microbial communities^40,41^. Both dead and dormant microbiome may still play a role in the microbiome by stimulating the production of immune molecules or releasing metabolites that can impact other members of the microbial community^37^. The complex interactions between active, dead, and dormant microbes should be examined in future studies. The use of network correlation-based analysis provides a comprehensive view of the complex interactions between viruses and bacteria, which would be difficult to assess with other methods. This helps to uncover correlations amongst microbes before subsequent detailed interrogation of specific networks can be carried out to identify factors that may be driving the assembly, function and stability of the microbiome. However, correlation analysis relies on the quality and completeness of the existing interactome data and often assume that associations are linear. The correlations identified should also not be interpretated as causal. Further validation using other clustering, as well as meta-analysis on a larger patient cohort should be carried out to confirm these microbial-level correlations.

Our study can serve as a springboard for future research to examine clinical applications of the unique alterations in virome associated with CRC. Bacteriophages may possibly be introduced into the gut to reverse microbial dysbiosis and serve as a “vaccine” for cancer. Future studies can also seek to explore the role of CRC-associated microbial biomarker in cancer screening and surveillance. Patients detected to have specific patterns of microbial dysbiosis associated with CRC might allow for a more timely diagnosis. Similarly, tracking compositional shifts in the microbiome can serve as an additional non-invasive adjunct in the surveillance of CRC following surgery.

In conclusion, the results of our study showed that microbial signatures characteristic of CRC include an altered virome besides an altered bacterial composition. Some patterns of dysbiosis persist even after surgery, suggesting the possibility of a pre-existing microbial field-change in the colon that predisposed to CRC in the first place. Correlations between bacteriophages and the bacteriome may imply that bacteriophages perhaps function as central nodes around which an oncogenic gut bacteriome forms, leading to carcinogenesis. Future studies should seek to verify if dysbiosis truly persists after surgery in a larger sample size with microbiome data collected at various time points after surgery, and if persistent dysbiosis correlates with patient outcomes.

### Supplementary Information

Supplementary Figure 1.

Supplementary Figure 2.

## Data Availability

The datasets generated and/or analysed during the current study are available in the BioProject repository, Accession number PRJNA905353 (https://dataview.ncbi.nlm.nih.gov/object/PRJNA905353?reviewer=amuubfuf2igulcv5rqkhvj74fp).
