# Supplementary figures and images for: Alterations in colorectal cancer virome and its persistence after surgery
Source: Sci Rep. 2024 Feb 3;14:2819. doi: 10.1038/s41598-024-53041-z (PMC10837111; doi:10.1038/s41598-024-53041-z)

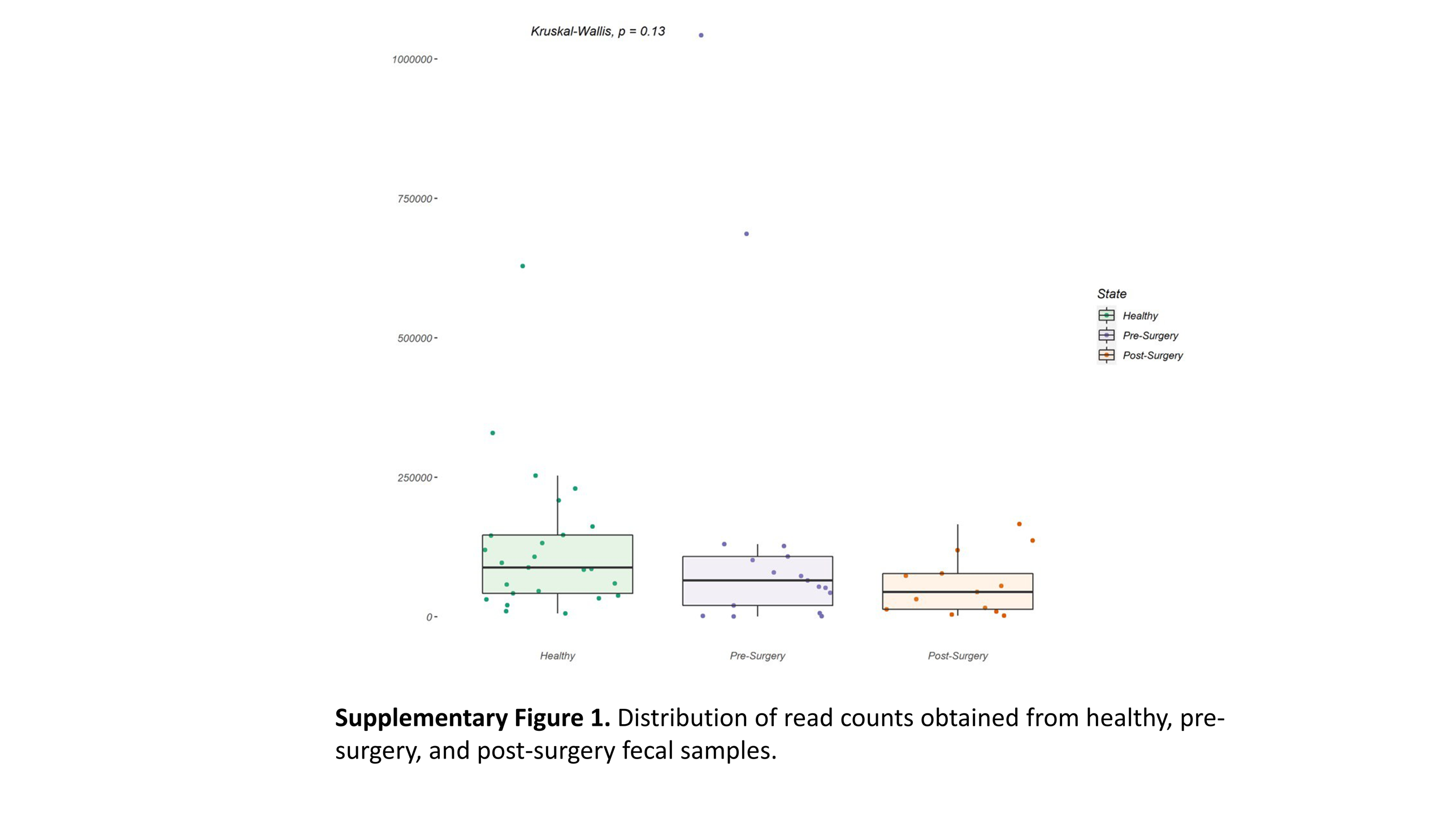

Supplement: Supplementary file 1 — Supplementary Figure 1. [file 41598_2024_53041_MOESM1_ESM.tif]

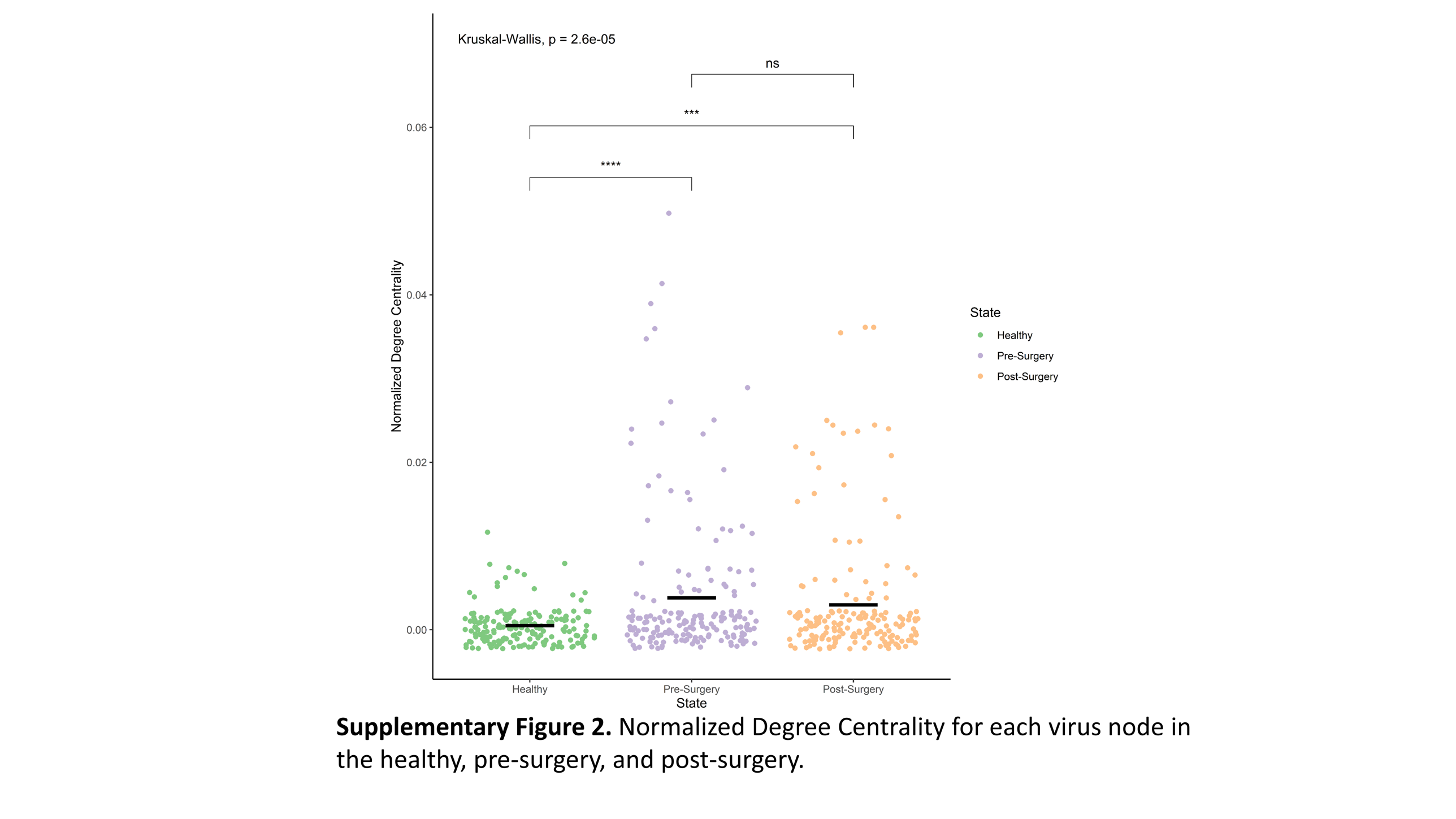

Supplement: Supplementary file 2 — Supplementary Figure 2. [file 41598_2024_53041_MOESM2_ESM.tif]
